# Evaluating the ability of a predictive vision-based machine learning model to measure changes in gait in response to medication and DBS within individuals with Parkinson’s disease

**DOI:** 10.1186/s12938-023-01175-y

**Published:** 2023-12-11

**Authors:** Andrea Sabo, Andrea Iaboni, Babak Taati, Alfonso Fasano, Carolina Gorodetsky

**Affiliations:** 1 KITE, Toronto Rehabilitation Institute, University Health Network, 550 University Avenue, Toronto, ON M5G 2A2 Canada; 2 Department of Psychiatry, University of Toronto, 250 College Street, 8th Floor, Toronto, ON M5T 1R8 Canada; 3 Centre for Mental Health, University Health Network, 33 Russell Street, Toronto, ON M5S 2S1 Canada; 4 Department of Computer Science, University of Toronto, 10 King’s College Road, Room 3302, Toronto, ON M5S 3G4 Canada; 5 Institute of Biomedical Engineering, University of Toronto, 164 College Street. Room 407, Toronto, ON M2S 3G9 Canada; 6 Vector Institute, 661 University Ave Suite 710, Toronto, ON M5G 1M1 Canada; 7 Division of Neurology, The Hospital for Sick Children, University of Toronto, 555 University Avenue, Toronto, ON M5G 1X8 Canada; 8 Edmond J. Safra Program in Parkinson’s Disease, Morton and Gloria Shulman Movement Disorders Clinic, Toronto Western Hospital, UHN, Toronto, ON Canada; 9 Krembil Brain Institute, Toronto, ON Canada; 10 CenteR for Advancing Neurotechnological Innovation to Application (CRANIA), Toronto, ON Canada

**Keywords:** Parkinson’s disease, Dementia, Gait analysis, Computer vision, Machine learning

## Abstract

**Introduction:**

Gait impairments in Parkinson’s disease (PD) are treated with dopaminergic medication or deep-brain stimulation (DBS), although the magnitude of the response is variable between individuals. Computer vision-based approaches have previously been evaluated for measuring the severity of parkinsonian gait in videos, but have not been evaluated for their ability to identify changes within individuals in response to treatment. This pilot study examines whether a vision-based model, trained on videos of parkinsonism, is able to detect improvement in parkinsonian gait in people with PD in response to medication and DBS use.

**Methods:**

A spatial–temporal graph convolutional model was trained to predict MDS-UPDRS-gait scores in 362 videos from 14 older adults with drug-induced parkinsonism. This model was then used to predict MDS-UPDRS-gait scores on a different dataset of 42 paired videos from 13 individuals with PD, recorded while ON and OFF medication and DBS treatment during the same clinical visit. Statistical methods were used to assess whether the model was responsive to changes in gait in the ON and OFF states.

**Results:**

The MDS-UPDRS-gait scores predicted by the model were lower on average (representing improved gait; *p* = 0.017, Cohen’s d = 0.495) during the ON medication and DBS treatment conditions. The magnitude of the differences between ON and OFF state was significantly correlated between model predictions and clinician annotations (*p* = 0.004). The predicted scores were significantly correlated with the clinician scores (Kendall’s tau-b = 0.301, *p* = 0.010), but were distributed in a smaller range as compared to the clinician scores.

**Conclusion:**

A vision-based model trained on parkinsonian gait did not accurately predict MDS-UPDRS-gait scores in a different PD cohort, but detected weak, but statistically significant proportional changes in response to medication and DBS use. Large, clinically validated datasets of videos captured in many different settings and treatment conditions are required to develop accurate vision-based models of parkinsonian gait.

**Supplementary Information:**

The online version contains supplementary material available at 10.1186/s12938-023-01175-y.

## Background

Parkinson’s disease (PD) is a neurodegenerative disorder that leads to deterioration of motor function [1]. There is a marked effect on gait, with forward-stooped posture, reduced range of motion, asymmetrical arm swing, and short shuffling steps commonly associated with the condition [2]. At advanced stages of PD, freezing of gait and balance disturbances become more common, greatly increasing the risk of falls [2, 3]. To manage the motor disturbances caused by PD and reduce their impact on an individual’s quality of life, dopaminergic medications and deep brain stimulation (DBS) therapies are commonly used [4]. The efficacy of these treatments varies over time within individuals, as well as between individuals, so timely monitoring and appropriate titration of therapies is essential [4].

It is often not possible for specialist clinicians to assess patients more than once or twice a year, so solutions that can be used to monitor motor symptoms outside of the clinic are needed to identify short-term fluctuations. The previous work has proposed the use of body or wrist-worn inertial measurement units (IMUs) to monitor tremors, dyskinesias, and gait of individuals with PD in clinical and residential settings [5–7]. Although wearable solutions are generally accurate in their measurements of parkinsonian motor symptoms, some concerns have been identified with respect to compliance when used outside of the clinic. In one study of adults with PD, a compliance of 62–68% (and decreasing during enrolment in the study) was noted when individuals were asked to wear a single wrist-worn device everyday [8]. Furthermore, wrist-worn devices are often insufficient to assess gait accurately, so trunk or ankle-worn IMUs are also required, thus further reducing the acceptability of this modality [5].

As an alternative, single-camera solutions have been proposed for assessment of gait quality in individuals with PD [9–11]. Camera-based solutions can be set-up either temporarily or permanently in group or individual residential settings, particularly in hallways where individuals generally walk in a straight path towards the camera [9, 12]. Previous work in this field has focused on using machine learning models to predict parkinsonism severity from video in accordance with the gait subscore of the Movement Disorders Society revision of the Unified Parkinson’s Disease Rating Scale (MDS-UPDRS-gait) [13]. The MDS-UPDRS-gait scale is an integer score between 0 and 4 (inclusive) where a higher score indicates gait with more severe parkinsonian characteristics [13]. Previous vision-based models are evaluated on their ability to predict the same MDS-UPDRS-gait score as a clinical annotator, with the existing models achieving promising results with F1-scores (harmonic mean of precision and recall) of 0.44–0.66 [9, 10] and balanced accuracies of 48–72% [10, 11].

However, further investigation is needed before vision-based gait assessment systems can be deployed for use by patients outside of the clinic in a meaningful way. The performance of previous systems has been evaluated solely on their accuracy in predicting whole-number (integer) MDS-UPDRS-gait scores [9–11]. The predictions of these previous systems have not been assessed for responsiveness to medication or DBS use. The effects of these treatments on gait quality can be visually observed by clinicians, so it is vital that vision-based systems are also able to reliably differentiate between on and off-treatment gait changes within an individual patient.

A second limitation of previous work is that models for assessing parkinsonism severity in gait have been trained and evaluated on the same dataset. Thus, we know little about how well a machine learning model will perform when predicting MDS-UPDRS-gait scores on videos of previously unseen patients collected in a new setting. Models have also typically been developed in a clinically homogenous population [ie only PD or only drug induced parkinsonism (DIP)]. For a vision-based solution to be valuable for nonresearch use, it is important that it be reliable in detecting change across different individuals, across a range of diagnoses associated with parkinsonian gait, and in a variety of settings.

This study aims to address these questions by evaluating the responsiveness of a machine learning model developed to recognize parkinsonian gait in DIP to the assessment of change in gait within a different cohort of individuals with PD when on medication and with their DBS device activated, compared to when they are off medication and their DBS device is deactivated. We evaluate whether the MDS-UPDRS-gait scores predicted by vision-based machine learning models are significantly lower when the participants are on treatment (medication and DBS) compared to when they are off treatment for walking bouts collected during the same clinical visit. Furthermore, we evaluate whether the magnitude of the difference between on and off states, as predicted by the models, are correlated with the differences noted by expert clinicians using the MDS-UPDRS-gait criterion. All models were trained on videos from one dataset and are evaluated on a different dataset to investigate how well they generalize to unseen data and whether the model is able identify features of parkinsonian gait common across diagnoses. Dopaminergic medications and DBS devices are used to manage parkinsonian symptoms in gait, but the efficacy of these treatments can vary and wane over time. Therefore, a machine learning model sensitive to when an individual’s gait begins to worsen and exhibit characteristics similar to untreated parkinsonism can be valuable to help advise clinicians whether a formal, in-clinic assessment is warranted.

## Results

### Prediction of MDS-UPDRS-gait scores

In total, 21 paired ON/OFF state walking bouts (42 total walking bouts) from 13 participants with PD were collected in the MDC. Table 1 describes the participants and available video data.

**Table 1:** Clinical information of participants and walking bouts by dataset

		Cohort		
		Training (DIP)	Testing (MDC)	
Number of participants		14	13	
Age (years)		76.2 ± 8.7	63.8 ± 8.8	
Sex (% male, *n*)		57.1%, 8 male	69.2%, 9 male	
PD duration (years)		N/A	10.8 ± 2.7	
MDS-UPDRS part III total score—median (range)		Unavailable	38 (17–66)	
Median and range of paired clinical visits per participant (total)		N/A	2 (range: 1–2, total pairs: 21)	
Number of videos by MDS-UPDRS part III gait subscore			Rater 1	Rater 2
	0	91	9	9
	1	111	16	18
	2	160	13	9
	3	0	4	6
	4	0	0	0
	Total	362	42	42

*DIP* dataset of older adults with drug induced parkinsonism, *PD* Movement disorders clinic dataset of adults with Parkinson’s disease, *MDS-UPDRS-III* Motor examination (part III) of the Movement Disorders Society revision of the Unified Parkinson’s Disease Rating Scale, *N/A* Not applicable, *PD* Parkinson’s disease

**Fig. 1:**
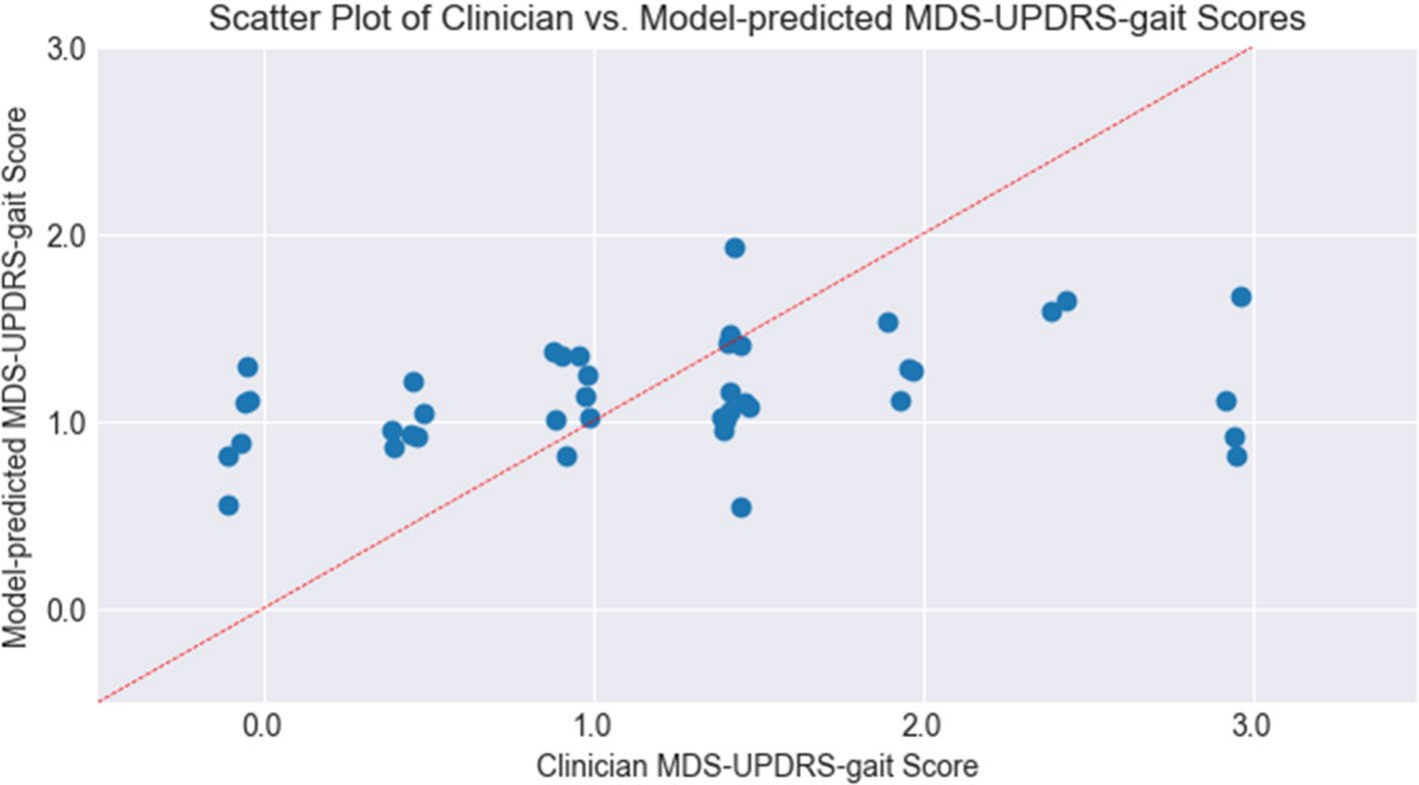
Scatter plot of clinician annotated vs ml model predicted MDS-UPDRS-gait score on all walking bouts. A small amount of jitter is applied to the clinician scores to facilitate clearer visualization of walks with the same score

Overall, the ML model trained on the DIP dataset had difficulty predicting the exact clinician rating for each walk in the PD dataset. The scatter plot in Fig. 1 does not show a clear correlation between the clinician annotations and the model predictions. The range of the MDS-UPDRS-gait scores predicted by the model is also smaller than the range of scores annotated by the clinicians, with most model-predicted values rounding to 1. This is also reflected in the low macro-averaged precision (0.40 ± 0.12), recall (0.30 ± 0.05), and F1-scores (0.22 ± 0.07) presented in Table 2. The overall unbalanced accuracy of the model was 33.3%, while the balanced accuracy was 29.5%.

**Table 2:** Macro-averaged precision, recall, f1-score, mean MDS-UPDRS-gait score prediction during on and off states and paired t-test significance value

Precision of rounded predictions (mean ± STD)	Recall of rounded predictions (mean ± STD)	F1-score of rounded predictions (mean ± STD)	Mean predicted MDS-UPDRS-gait score—OFF treatment	Mean predicted MDS-UPDRS-gait score—ON treatment	Paired t-test p-value for difference in model predicted scores in ON/OFF states	Mean clinician assessed MDS-UPDRS-gait score—OFF treatment	Mean clinician assessed MDS-UPDRS-gait score—ON treatment	Paired t-test p-value for difference in model clinician assessed scores in ON/OFF states
0.40 ± 0.12	0.30 ± 0.05	0.22 ± 0.07	1.22 ± 0.32	1.07 ± 0.24	0.017	1.71 ± 0.86	0.86 ± 0.67	< 0.001

The standard deviation represents the magnitude of the variation between the five repetitions of the experiments

*MDS-UPDRS-gait* Gait subscore (item 3.10) of the Movement Disorders Society revision of the Unified Parkinson’s Disease Rating Scale, *STD* Standard deviation

A Kendall’s Tau-b correlation coefficient (τ_B_) of 0.301 (*p* = 0.004, one-tailed) was calculated between the discrete MDS-UPRDS-gait scores annotated by the clinicians and the continuous scores predicted by the model. These results suggest a weak but statistically significant correlation between the mean clinician scores and the model predictions.

### ICC between clinician annotations and model-predicted MDS-UPDRS-gait scores

The ICC between the two clinicians’ ratings was 0.73 (CI 95% = [0.54, 0.84], *p* < 0.001). The ICC between the model-predicted MDS-UPDRS-gait scores and the mean clinician-annotated scores was 0.19 (*p* = 0.112, 95% CI [−0.12, 0.46]). The ICC between the model predictions and Rater 1 (the unblinded rater) was 0.18 (*p* = 0.120, 95% CI [−0.12, 0.46]), while the ICC between the model predictions and Rater 2 (the blinded rater) was 0.31 (*p* = 0.023, 95% CI [0.01, 0.56]). The calculation of ICC requires the comparison of values belonging to discrete classes, thus relying on the rounding of model-predicted values to the closest integer to match with the scores annotated by the clinicians. Due to the smaller range of values predicted by model, most values were rounded to 1, and yielded a low overall ICC when comparing model predictions to clinician annotations. A complete analysis of performance by clinician is presented in Additional file 1: Appendix E.

### Detection of change in MDS-UPDRS-gait score in ON and OFF state

The mean clinician gait scores were significantly lower on average in the ON states (0.86 ± 0.67) compared to OFF states (1.71 ± 0.86, *p* < 0.001; Cohen’s d (effect size) = 1.21, 95% CI [0.65–1.81]; Table 2). The model-predicted scores also showed responsiveness to treatment, with predicted lower scores on average in the ON state (1.07 ± 0.24) compared to OFF state (1.22 ± 0.32, *p* = 0.017; Cohen’s d (effect size) = 0.50, 95% CI [0.04–0.97]; Table 2). Due to the continuous and smaller range of the model-predicted MDS-UPDRS-gait scores, a cut-off of 0.15—the difference in mean predicted score in the ON and OFF states was used to identify if the model predicted improved, worsened, or unchanged scores.

**Fig. 2:**
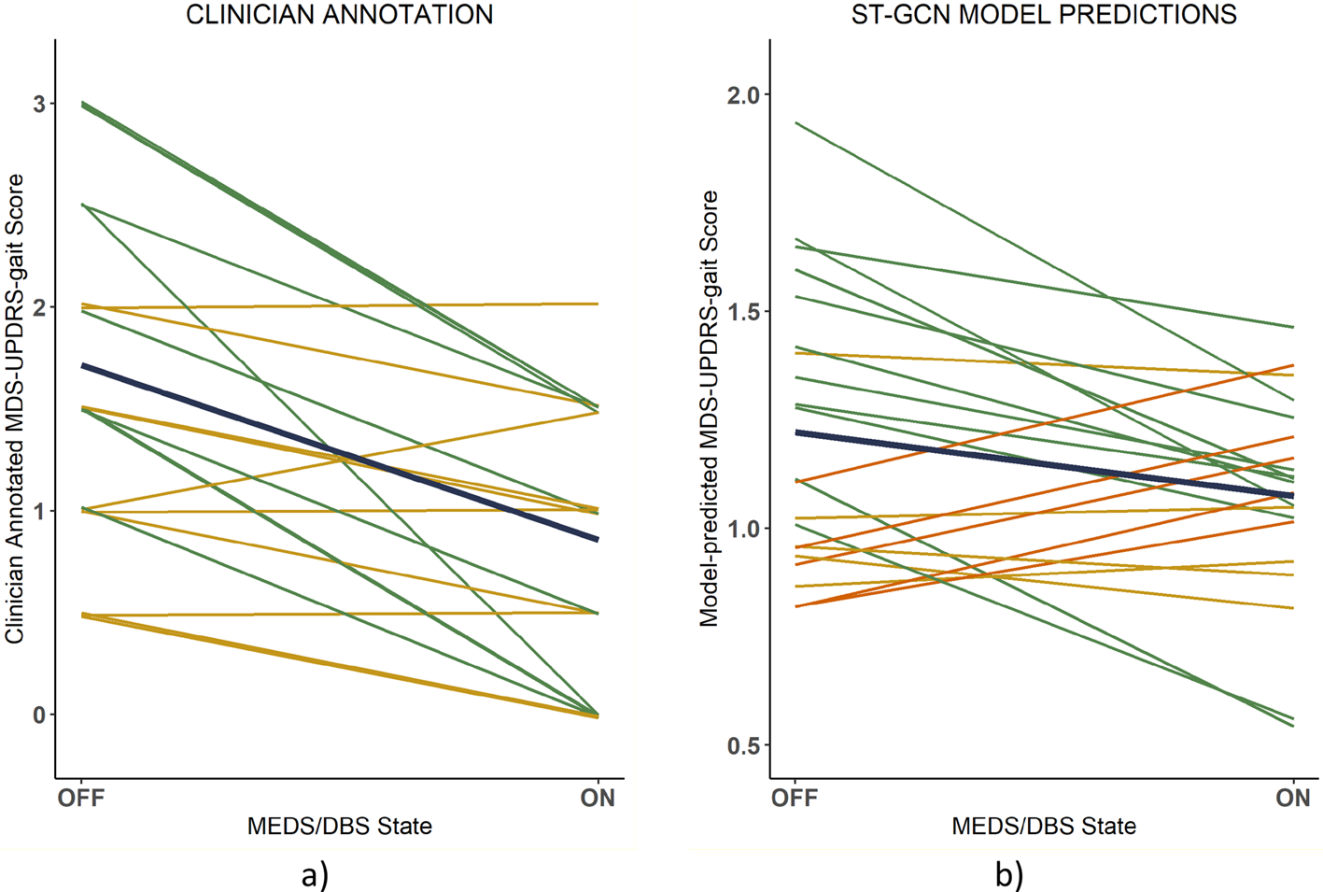
Spaghetti plots of MDS-UPDRS-gait scores labelled by the clinician (**a**) and model (**b**) in on and off states, grouped by patient and clinical visit. Green lines indicate paired walks where the mds-updrs-gait score was higher in the off state than the on state (indicating improvement in gait on treatment), while red lines denote the pairs where the mds-updrs-gait score was higher on treatment (indicating worsening gait). Yellow is used to denote pairs where no change was noted between the two treatment conditions. The navy lines represent the mean prediction for each treatment condition. Note that a small random jitter factor was applied to each clinician annotated score to improve the visualization

In 11 paired ON/OFF state walks, the mean clinician-rated gait scores improved (were lower) by at least 1 point on the MDS-UPDRS-gait scale during the ON state compared to the OFF state (Fig. 2). In 10 of 21 paired walks, the difference between the mean clinician MDS-UPDRS-gait score in the two conditions was 0.5 or less, indicating no improvement or that the improvement was not noted by both clinicians (who were restricted to rating using integer values). Conversely, the ML model predicted improved (lower) MDS-UPDRS-gait scores in the ON state when it was tested on the PD dataset in 11 paired assessments, unchanged scores 5 pairs, and worsened scores in the ON state of 5 paired assessments (Fig. 2).

Predictions of whether there was an improvement/no change/worsening of MDS-UPDRS-gait score in ON/OFF states were congruent between the clinician and model in 62.0% of paired assessments. The two clinicians were congruent in their assessment of whether there was an improvement/no change/worsening in MDS-UPDRS-gait scores in 66.7% of the paired assessments. Assessments where the model failed to detect clinician-noted improvement in gait with treatment tended to be when the clinician rating in the OFF condition was higher. Specifically, the model predictions for improvement were only congruent with the clinician in 5 of the 9 assessments when the mean clinician rating in the OFF state was between 2 and 3, inclusive. Conversely, the model and clinicians were aligned in their predictions of improved vs unchanged/worsened MDS-UPDRS-gait scores in 8 of the 12 walks when the mean clinician assessment in the OFF state was between 0 and 1.5, inclusive.

To assess the reliability and stability of the model predictions, the direction of change of the model predictions and agreement to clinician ratings was assessed for each of the 5 training repetitions in Additional file 1: Appendix D. Based on this analysis, we note that the model can reliability detect change in MDS-UPDRS-gait score between treatment conditions, even though it has difficulty predicting the absolute score.

### Magnitude of change ON/OFF treatment

Kendall’s Tau-b correlation coefficient (τ_B_) for correlation of the difference in MDS-UPDRS-gait scores as labelled by the clinicians and by the model was 0.396 (*p* = 0.010, one-tailed). Therefore, the magnitude of the difference across the ON and OFF state scores was significantly correlated between clinician and model predictions. This indicates that when the clinicians note a larger difference in MDS-UPDRS-gait score between the ON and OFF state for paired walks in the same clinical visit, the scores predicted by the model also have a larger difference.

**Fig. 3:**
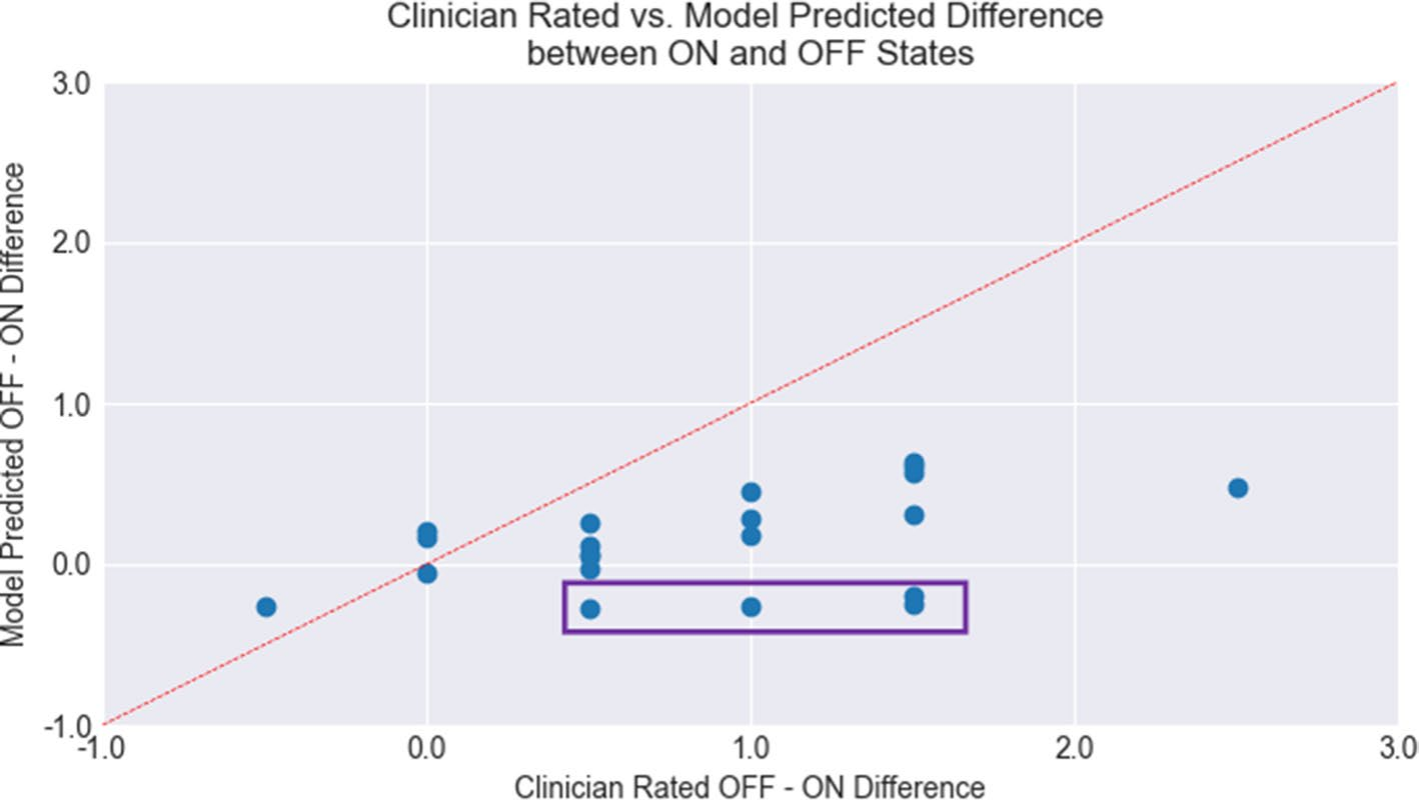
Scatterplot of difference in MDS-UPDRS-gait score as predicted by the model and labelled by the clinician in off and on states. The points in purple box correspond to the 4 trials in which the model noted worsening mds-updrs-gait scores between the off and on treatment states that were not observed by the clinicians

Figure 3 presents a scatterplot of the differences in model predicted MDS-UPDRS-gait scores in OFF and ON states, as grouped by the difference in scores noted by the clinicians. This figure visualizes that the difference in scores in the OFF and ON states is correlated between clinician annotations and model predictions.

## Discussion

In this work, we used a machine learning model trained to predict MDS-UPDRS-gait scores in older adults with DIP to evaluate change in parkinsonian gait in participants with PD in response to treatment.

Our results indicate that the machine learning model struggled to transfer its ability to predict absolute MDS-UPDRS-gait scores across datasets, with a lower precision, recall, and F1-score than those noted in our previous within-dataset model evaluation [9]. However, when comparing the difference in scores predicted during ON and OFF states during the same clinical visits, the predicted scores in the ON state were generally lower than those for the OFF state when the ML model was used. These findings suggest that while the ML model is unable to accurately capture differences in parkinsonism severity between participants, likely due to the variation in gait between individuals, the model is able to capture relative differences between paired walks from the same individual. Although the model is not accurate in its prediction of the absolute value of the clinician rated MDS-UPDRS-gait scores on the unseen dataset, the scores it predicts show responsiveness as the model is able to identify changes in parkinsonism severity in gait associated with medication and DBS use. Therefore, there may be the potential to leverage future iterations of this model by comparing the predicted scores over longitudinal measurements and assessing the change over time within a patient. For example, if an individual experiences a consistent increase in their model-predicted MDS-UPDRS-gait score over the course of several weeks or months, it may indicate that an adjustment to their symptom management plan is required, and a formal clinical evaluation should be scheduled.

It is important to note that the changes in MDS-UPDRS-gait scores predicted by the model are lower than the changes in scores when calculated from clinician annotations. However, the magnitude of change between ON and OFF state scores predicted by the model is correlated with the change in scores between the two states as calculated from clinician annotations (Fig. 3). Based on these findings, the proposed ML model operating on joint trajectories extracted using the Detectron pose-estimation library can capture the direction and magnitude of change in MDS-UPDRS-gait scores within a patient similarly to a clinician annotator.

To the best of our knowledge, this is the first work to use a vision-based model to assess changes in predicted scores of parkinsonism in gait during ON and OFF states during the same clinical visit. In related work, engineered features extracted using pose-estimation models have been successfully used to detect the onset and remission of levodopa-induced dyskinesia in individuals with PD from video [14]. In the field of gait analysis, work by other groups has investigated the accuracy of vision-based systems in predicting the same MDS-UPDRS-gait scores as clinicians, but has not evaluated whether the systems were sensitive to changes in medication or DBS within an individual [10, 11]. Work by Rupprechter and Morinan et al. demonstrated a significant difference in five gait features between “on medication” and “off medication” states, but did not evaluate whether the MDS-UPDRS-gait scores predicted by their final model were sensitive to medication or DBS use [11]. In contrast, our study is the first to evaluate the performance of a vision-based MDS-UPDRS-gait prediction system in paired walks to assess changes by participant, a use-case that more closely represents a situation where an individual would use the system to monitor changes in their own gait (rather than that of a group) over time.

Furthermore, this is the first work to evaluate how well a model trained for MDS-UPDRS-gait prediction on one dataset transfers to a different, independently collected, dataset. Training on a cohort of older adults with DIP, we have shown that an appropriate model and pose-estimation library can be used to identify changes in parkinsonism severity within a person with PD on an unseen dataset. However, the absolute scores predicted by our model are not calibrated or strongly correlated with the clinician’s ratings, resulting in a poor overall model accuracy. For this reason, score changes should only be compared within a participant, rather than between participants or between clinician labels and model predictions.

Frequent evaluations of the gait severity in Parkinson’s disease patients are clinically very important. Typical gait manifestations include reduced speed and step length, increased axial rigidity and impaired rhythmicity. With disease progression gait problems tend to worsen and affect independence and quality of life [2]. Dopaminergic medications and DBS are the mainstream treatments for PD. Despite the overall good effect of these modalities on motor manifestations of PD, their specific effect on gait tends to be more variable and may lead, in some patients, to aggravation of freezing of gait [15]. Furthermore, some individuals might experience delayed gait worsening, highlighting the need for a continuous assessment. Specific DBS parameters (as low frequency stimulation) were found to be more beneficial to improve gait abnormalities in this population, hence the need for reliable and sensitive ML methods to capture these clinical changes.

One limitation of this study is that one of the clinicians was not blinded to the ON/OFF status of the participants which may have also biased the score assigned. A previous study has noted that there was moderate variability in MDS-UPDRS part III gait score annotations when rated by multiple clinicians (ICC = 0.746) [16]. This is similar to the ICC of 0.73 noted between the two clinicians’ ratings on the PD dataset in this study. The performance of the model when compared to each clinician separately is presented in Additional file 1: Appendix E.

Training models on data from multiple annotators could thus be used to provide additional confidence and robustness, particularly for walks for which the MDS-UPDRS-gait score may be ambiguous. Furthermore, the number of individuals on which the model was trained and evaluated (14 and 13 participants respectively) was limited due to data availability. Moreover, the training dataset did not include any examples of severe MDS-UPDRS-gait ratings while the test cohort had several walks with clinical scores of 3. The lack of highly impaired data in the training dataset is a potential reason why the model struggled to identify improvement in more impaired walks when evaluated on the test cohort. To address this limitation, an experiment where the model was trained on data from both the DIP and PD datasets was performed. Using the whole DIP cohort and the PD dataset in a leave-on-subject-out cross-validation scheme, separate models were trained to predict the MDS-UPDRS-gait scores for each participant in the PD cohort. While it was hypothesized that the addition of dataset specific training data would improve the sensitivity of the models, this behavior was not observed. Instead, the inclusion of training data from datasets with two different populations, data collection methodologies, and annotated by two different raters provided conflicting information to the model during training, preventing it from learning a consistent representation of each score. Detailed results and further comments about this experiment are available in Additional file 1: Appendix B.

Additionally, this study relied on the use of videos taken from a coronal view. The model was trained on the DIP dataset where participants walked down a hallway while a camera attached to the ceiling recorded their gait. The position and orientation of this camera was fixed for all walks recorded as part of this dataset. In contrast, during data collection for the MDC dataset, the tripod-mounted camera was positioned to the side of the instrumented gait mat on which the participants walked, but its position and orientation were not strictly controlled between walks and participants. A diagram and sample frame from this data collection methodology is available in [17], demonstrating the slight angle of the camera with respect to the direction of movement. While the lack of standardization in the camera position and orientation with respect to the direction of movement is similar to a potential real-world data collection environment, this also increased the variance in the input (as compared to the DIP dataset) and thus likely the output predictions as well. While this study only evaluated videos taken from a primarily coronal view, however certain aspects of gait such as those associated with step length or speed are more reliably captured from a sagittal view [18]. However, sagittal views generally require more physical space to take which is not always feasible in a home environment, whereas coronal view videos can be taken in hallways commonly found in home-living environments [19].

Another limitation of this work is that a dataset of older adults with dementia and DIP was used to train the model, unlike the dataset of individuals with PD on which the model was evaluated. Previous work comparing the gait of individuals with DIP and PD during the Timed Up & Go (TUG) test found that while there were significant differences in the rotation time, anterior–posterior step correlation, and medio-lateral sway, the straight walking time, cadence, step length, and medio-lateral step correlation was similar amongst the cohorts [20]. However, DIP is more commonly associated with symmetric parkinsonism, with 30–50% of individuals presenting with asymmetric parkinsonism which is more commonly associated with idiopathic PD [21]. In work by Yahalom and Israeli-Korn et al., difference in left–right asymmetry was borderline significant (*p* = 0.06) in age-matched DIP and PD cohorts [20]. It has also been noted that this difference in DIP and PD is more prevalent in younger patients, whereas individuals with PD and older adults with DIP generally present with similar motor symptoms [21]. However, it is important to acknowledge that different populations were used for model training and evaluation in this study. Finally, the study was not designed to capture the differences between dopaminergic medication and DBS when used alone and not in combination.

Future work will focus on strengthening the predictive ML model by better calibrating the scores it predicts with the MDS-UPDRS-gait scale. Currently, the proposed model is limited in its generalizability as it was trained on a relatively small number of videos from a DIP population taken from one camera angle and annotated by one clinician. As seen in this study, the shift to a different population in which videos were taken from different viewpoints and annotated by different clinicians led to model predictions which were not calibrated to the clinical annotations. Therefore, a more robust model that has been trained on a wider and more varied set of input data is needed. This will require the collection of additional videos of individuals with idiopathic PD, as well as MDS-UPDRS-gait annotations from multiple raters for each video to provide more robust training data for our model. There is also the opportunity to record walking bouts from multiple planes of view as a means of data augmentation during training, and to simulate different testing environments during evaluation. Additionally, the collection of multiple walking bouts of each individual under the same treatment condition in close succession would allow for the assessment of the model’s reliability. A reliable model for assessing parkinsonism in gait should predict MDS-UPDRS-gait scores within a small range when walks are recorded a few minutes apart (without any change in treatment). Furthermore, we hope that by closely calibrating the model outputs to the MDS-UPDRS-gait standardized scale, it will be possible for clinicians to interpret predicted scores more meaningfully from different participants, without having to rely on relative changes within a participant’s walks. Additional experiments on walking bouts of adults with PD collected longitudinally in home settings (ie. between clinical visits) are needed to evaluate whether the machine learning model is able to detect daily fluctuations in parkinsonism severity. Finally, newer ML model architectures such as transformers will be evaluated for their applicability to this longitudinal MDS-UPDRS-gait estimation task [22].

## Conclusion

A model trained on videos of older adults with DIP was sensitive to changes in medication and DBS use in a different dataset of videos of adults with PD, although not accurate at predicting the exact clinical score, and had difficulty detecting improvement in those with severely impaired gait. These results suggest that appropriate vision-based machine learning models are presently not accurate enough to become the standard of care but have potential to be used to monitor fluctuations in gait in individuals with PD in future iterations. Future work will focus on more closely calibrating scores predicted by the model with the MDS-UPDRS-gait criteria.

## Methods

### PD data collection

Participants were adults with idiopathic PD at an academic movement disorders clinic (MDC) who were treated with dopaminergic medication and had an implanted subthalamic nucleus (STN)-DBS device. The DBS stimulation parameters were determined by the treating neurologist. As part of a longitudinal study, each participant was assessed in an OFF and ON treatment state during multiple clinical visits. In the OFF state, the participant’s DBS device was turned off and they had not taken dopaminergic medication for at least 12 h before assessment (‘practically defined off’). Conversely, the ON state was assessed 45–60 min after the participant had taken their prescribed dose of dopaminergic medication and their DBS device had been turned on. Patients who were treated with medications only or DBS only were excluded from the study.

A tripod-mounted camera (640 × 480 resolution, sampling rate of 30 Hz) was used to record participants’ gait during each clinical visit. The participants were instructed to walk 6 m, turn around, and then walk back to their starting position. As the model was developed on video data of people walking towards the camera, only the sections of the video where the participants were walking toward the camera were included. Two physicians affiliated (CG, AI) with the study scored each walking bout on the MDS-UPDRS-gait criteria from the recorded videos independently. As one clinician was present during data collection, they were not blinded to participant ON/OFF state during scoring. Conversely, the other clinician was blinded to ON/OFF state. The Research Ethics Board of the University Health Network approved the protocol (17-5707; March 1, 2018) and all study participants provided informed written consent.

### Calculation of intraclass correlation coefficient for clinician annotations

The agreement between the two clinician annotators was assessed by calculating the intraclass correlation coefficient (ICC). The ICC(3), which assesses the agreement between two raters who both scored the full dataset, was calculated using the pinguin library for Python [23].

### Model for prediction of MDS-UPDRS-gait scores from video

A method for predicting MDS-UPDRS-gait scores from coronal videos was developed as part of previous work on a dataset of 362 walking bouts from 14 participants with varying severities of drug-induced parkinsonism (DIP) [24–27]. To develop this model, three open-source human pose-estimation libraries (AlphaPose, Detectron, OpenPose) were first used to predict the locations of key body joints in each frame of the videos [28–31]. Using these joint trajectories as input, we trained a spatial–temporal graph convolutional network (ST-GCN) machine learning (ML) model to predict the severity of DIP in gait [9]. ST-GCN models are extensions of graph convolutional models proposed by Kipf and Welling [32] in which convolutions are applied in both spatial and temporal dimensions, taking into account the structure of the data and how joints are connected to each other [33]. For example, in the first layer of the spatial convolution, joints that are immediately connected will be considered in the adjacency matrix, preserving the skeletal structure of the data. Work by other groups has also evaluated the use of ST-GCN models for the analysis of parkinsonism in gait [34, 35]. The full details of the model used in this study and its validation are described in detail in previous work by our group [9].

This model was trained on the DIP dataset was used to predict continuous MDS-UPDRS-gait scores on the PD dataset. The inputs to the model are joint trajectories for each walking bout as well as the clinician annotated integer MDS-UPDRS-gait scores. Joint trajectories from all three pose-estimation libraries were used during training, but the results for model evaluation are presented for data extracted by the Detectron library. The data extracted using this pose-estimation library was found to perform the best across datasets. Full results from the AlphaPose and OpenPose libraries are presented in Additional file 1: Appendix A. The architecture of the ML model allows it to predict a continuous (non-rounded) value of the MDS-UPDRS-gait score for each walking bout, which provides more granularity than integer predictions. To account for performance fluctuations due to known random processes during training, such as weight initialization or the order in which the data is input to the network, the model was trained five times and the mean prediction across all repetitions was used when reporting the model performance. The standard deviation across folds is also presented when appropriate. To improve the robustness of the model when transferring across datasets, the input data to the ML model was normalized by the mean hip to shoulder distance in each frame to account for different heights and video resolutions. This is a deviation from previous work where normalization of the skeletons was not performed [9].

### OF-DDNet

In addition to the ST-GCN model proposed in [9], an ordinal focal neural double-feature, double-motion network (OF-DDNet) proposed by Lu et al. [36] was also evaluated for this task. The OF-DDNet model did not achieve statistical significance when evaluating sensitivity to ON/OFF state on the PD test set and will thus not be discussed in detail in this manuscript. For full results on this model, please refer to Additional file 1: Appendix C.

### Statistical analysis

#### Prediction of rounded MDS-UPDRS-gait scores

To understand how well the trained ML model predicts MDS-UPDRS-gait scores on the PD dataset, the macro-averaged precision, recall, and F1-scores were calculated. The F1-score is calculated as the harmonic mean of precision and recall. The macro-average is calculated by computing the statistic of interest for each class and then taking the arithmetic mean of these per-class values. This ensures that each class is weighted equally in the final metric.

Furthermore, the unbalanced accuracy was calculated as the percentage of walks where the clinician-annotated score was the same as the model prediction (when rounded to the nearest integer). The balanced accuracy assigns an equal weighting to each clinician-assigned MDS-UPDRS-gait score, ensuring that the value is not influenced by class imbalance.

When determining the clinician reference score for these metrics, the mean of the scores assigned by each of the two physicians was calculated and rounded to the nearest integer and away from zero in case of ties.

#### Sensitivity of models to ON/OFF treatment state

To determine whether the continuous MDS-UPDRS-gait score predicted by the model was significantly lower in the ON state than the OFF state, a one-tailed paired t test was used. Walking bouts were paired by participant and clinical visit, and a Shapiro − Wilk test of normality was performed on the difference in predicted scores to validate the t test assumptions.

#### Magnitude of change between ON/OFF treatment-model vs. clinician rating correlation

A second analysis was performed to determine whether the difference in MDS-UPDRS-gait score between the ON and OFF states was correlated when model predictions and clinician rating were compared. The mean score assigned by the two clinicians for each walk (without rounding to integer scores) was used as the clinician annotation. For each clinical visit, the ON state MDS-UPDRS-gait score was subtracted from the OFF state score. This calculation was performed independently for the model predictions and the clinician annotations.

Kendall’s Tau-b test was used to correlate the model (continuous) and clinician (discrete) differences. All statistical analyses were performed using R (version 4.1.2) and the threshold for statistical significance was selected as *p* < 0.05 for all experiments.

### Supplementary Information

**Additional file 1: Appendix A.** Evaluation of Different Pose-Estimation Libraries. **Appendix B.** Training on both the DIP and PD Dataset in LOSOCV. **Appendix C.** Evaluation of OF-DDNet Model. **Appendix D.** Evaluation ST-GCN by Repetition. **Appendix E.** Evaluation by Clinician Rater

## Data Availability

The datasets generated and/or analyzed during the current study are not publicly available due to the identifying nature of the video recordings.
