# Supplementary material for: Evaluating the ability of a predictive vision-based machine learning model to measure changes in gait in response to medication and DBS within individuals with Parkinson’s disease
Source: Biomed Eng Online. 2023 Dec 11;22:120. doi: 10.1186/s12938-023-01175-y (PMC10714555; doi:10.1186/s12938-023-01175-y)
Supplement: Supplementary file 1 — Additional file 1: Appendix A. Evaluation of Different Pose-Estimation Libraries. Appendix B. Training on both the DIP and PD Dataset in LOSOCV. Appendix C. Evaluation of OF-DDNet Model. Appendix D. Evaluation ST-GCN by Repetition. Appendix E. Evaluation by Clinician Rater [file 12938_2023_1175_MOESM1_ESM.pdf]

Additional File 1

Appendix A – Evaluation of Different Pose-Estimation Libraries

In this study, three open-source libraries (AlphaPose, Detectron, OpenPose) were investigated for extracting joint locations from video. In previous work, we identified that data augmentation can be performed by extracting joint trajectories from different pose-estimation libraries and using all data during the training process. This data augmentation technique improved performance of the proposed ST-GCN ML model when evaluating on test data [1]. However, in an inference scenario, it is not feasible to pose-track all videos with multiple pose-estimation libraries due to the large computational requirements, so performance on the test set was evaluated separately for walk trajectories extracted from each library.

Results

Table A-1 presents the macro-averaged precision, recall, F1-score; as well as the mean predicted MDS-UPDRS-gait score in the ON and OFF states as predicted by the ST-GCN ML model presented in the main manuscript. Table A-2 presents the Kendall τ_B estimates and p-values for the correlations between the model-predicted and clinician annotated scores, as well as for the correlations between the differences in ON and OFF states as rated by the model and clinicians. Figure A-1 displays the trends in model-predicted MDS-UPDRS-gait scores in the ON and OFF states when paired by participant and clinical visit.

TABLE A-1

Macro-averaged Precision, Recall, F1-Score, Mean MDS-UPDRS-Gait Score Prediction During ON and OFF States and Paired T-Test Significance Value

Pose-estimation library	Precision of Rounded Predictions (mean ± STD)	Recall of Rounded Predictions (mean ± STD)	F1-score of Rounded Predictions (mean ± STD)	Mean predicted MDS-UPDRS-gait score – OFF treatment	Mean predicted MDS-UPDRS-gait score – ON treatment	Paired t-test p-value for difference in model predicted scores in ON/OFF states
**AlphaPose**	0.34 ± 0.14	0.29 ± 0.03	0.20 ± 0.05	1.32 ± 0.46	1.16 ± 0.47	**0.011**
**Detectron**	0.40 ± 0.12	0.30 ± 0.05	0.22 ± 0.07	1.22 ± 0.32	1.07 ± 0.24	**0.017**
**OpenPose**	0.39 ± 0.07	0.27 ± 0.02	0.20 ± 0.03	1.16 ± 0.46	1.03 ± 0.43	**0.041**

TABLE A-2

One-tailed Kendall τ_B Estimates and P-Values for Correlation Strength Between Model-Predicted and Clinician-Annotated MDS-UPDRS-Gait Values and Differences in Scores Between ON and OFF States

Pose-estimation library	Correlation of MDS-UPDRS-gait score predicted by ML model and annotated by clinicians		Correlation of difference between ON/OFF state as rated by clinicians and ML model	
	Kendall τ_B estimate	Kendall τ_B p-value	Kendall τ_B estimate	Kendall τ_B p-value
**AlphaPose**	0.25	**0.015**	0.12	0.237
**Detectron**	0.30	**0.004**	0.40	**0.010**
**OpenPose**	0.30	**0.004**	0.23	0.090

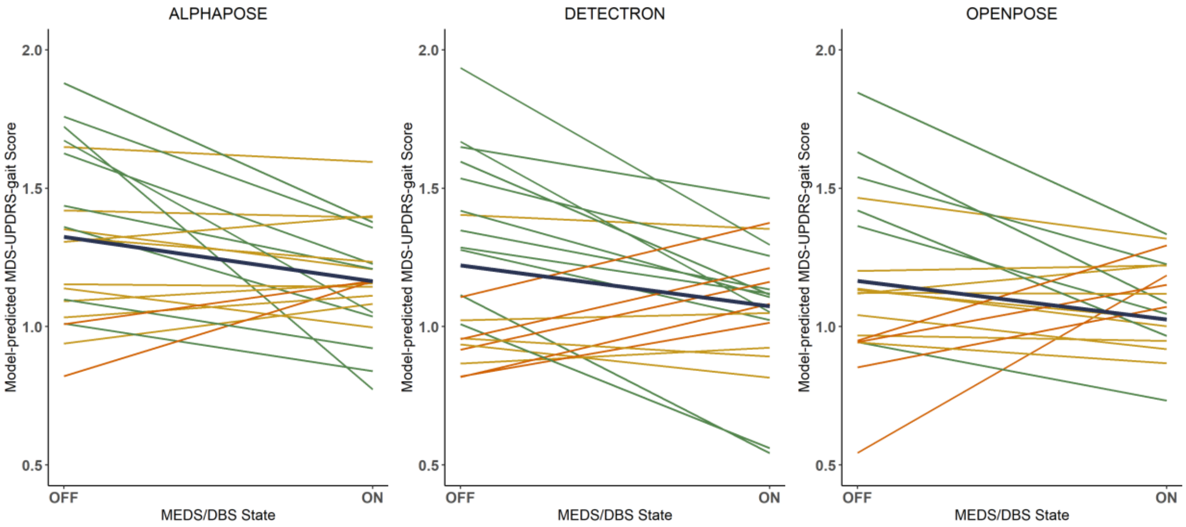

Figure A-1. Spaghetti plots of MDS-UPDRS-gait scores predicted by the model in ON and OFF states, grouped by patient and clinical visit. The red lines represent the mean prediction for each treatment condition.

Additional File 1

In our experiments, there was a significant difference in the mean MDS-UPDRS-gait score predicted by the model in the ON and OFF conditions when evaluating with each of the three pose-estimation libraries (Table A-1). The Kendall τ_B coefficient is moderate and statistically significant for the correlation between the discrete clinician-annotated scores and the continuous ML model predictions for all three pose-estimation libraries (Table A-2). However, the difference in the magnitude of change in ON and OFF states as rated by the clinicians and predicted by the model is only statistically significant for the Detectron pose-estimation library.

Conclusion

When evaluating the ST-GCN ML MDS-UPDRS-gait prediction model on the PD dataset available for this study, the Detectron library yielded the most statistically significant results for the metrics assessed.

Appendix B – Training on both the DIP and PD Dataset in LOSOCV

The main manuscript assessed the performance of the proposed ML model in predicting MDS-UPDRS-gait scores on an unseen PD cohort after being trained solely on a dataset of individuals with DIP. A further experiment was conducted to evaluate whether the addition of data from the PD dataset in the training set would improve performance.

Methods

The same ST-GCN ML model described in the main manuscript was trained on the entire DIP cohort and the PD cohort in a leave-one-subject cross-validation (LOSOCV) scheme, ensuring that the individual on whom the model was being evaluated was not included in the training data. The same training parameters, data normalization, and data augmentation approaches were used as for the models that were trained on only the DIP dataset.

Results

Table B-1 presents the macro-averaged precision, recall, F1-score; as well as the mean predicted MDS-UPDRS-gait score in the ON and OFF states on the test set of the model trained on the combined DIP and PD datasets (in a LOSOCV manner). Table B-2 presents the Kendall τ_B estimates and p-values for the correlations between the model-predicted and clinician annotated scores, as well as for the correlations between the differences in ON and OFF states as rated by the model and clinicians. Figure B-1 displays the trends in model-predicted MDS-UPDRS-gait scores in the ON and OFF states when paired by participant and clinical visit.

TABLE B-1

Macro-averaged Precision, Recall, F1-Score, Mean MDS-UPDRS-Gait Score Prediction During ON and OFF States and Paired T-Test Significance Value for the ST-GCN model trained on the DIP and PD Dataset in a LOSOCV Manner

Pose-estimation library	Precision of Rounded Predictions (mean ± STD)	Recall of Rounded Predictions (mean ± STD)	F1-score of Rounded Predictions (mean ± STD)	Mean predicted MDS-UPDRS-gait score – OFF treatment	Mean predicted MDS-UPDRS-gait score – ON treatment	Paired t-test p-value for difference in model predicted scores in ON/OFF states
**AlphaPose**	0.19 ± 0.11	0.20 ± 0.09	0.19 ± 0.10	1.78 ± 0.51	1.72 ± 0.55	0.078
**Detectron**	0.16 ± 0.10	0.17 ± 0.07	0.16 ± 0.08	1.72 ± 0.49	1.66 ± 0.53	0.109
**OpenPose**	0.13 ± 0.08	0.14 ± 0.05	0.13 ± 0.06	1.71 ± 0.48	1.63 ± 0.57	**0.050**

TABLE B-2

One-tailed Kendall τ_B Estimates and P-Values for Correlation Strength Between Model-Predicted and Clinician-Annotated MDS-UPDRS-Gait Values and Differences in Scores Between ON and OFF States for the ST-GCN model trained on the DIP and PD Dataset in a LOSOCV Manner

Pose-estimation library	Correlation of MDS-UPDRS-gait score predicted by ML model and annotated by clinicians		Correlation of difference between ON/OFF state as rated by clinicians and ML model	
	Kendall τ_B estimate	Kendall τ_B p-value	Kendall τ_B estimate	Kendall τ_B p-value
**AlphaPose**	-0.36	0.999	0.18	0.137
**Detectron**	-0.39	1.000	0.05	0.389
**OpenPose**	-0.41	1.000	-0.03	0.562

Additional File 1

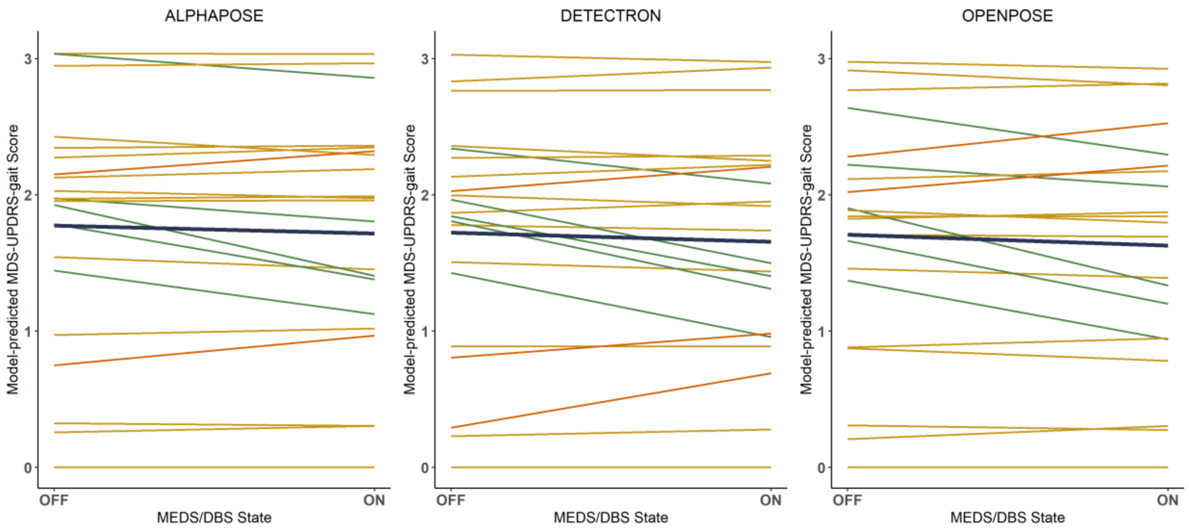

Figure B-1. Spaghetti plots of MDS-UPDRS-gait scores predicted by the model in ON and OFF states, grouped by patient and clinical visit. Green lines indicate paired walks where the MDS-UPDRS-gait score was higher in the OFF state than the ON state (indicating improvement in gait ON treatment), while red lines denote the pairs where the MDS-UPDRS-gait score was higher ON treatment (indicating worsening gait). Yellow is used to denote pairs where no change was noted between the two treatment conditions. The navy lines represent the mean prediction for each treatment condition.

Comparing Table B-1 to Table A-1, Table B-2 to Table A-2, and Figure B-1 to Figure A-1, it is evident that the addition of the training data from the PD dataset yielded poorer results when evaluating on the unseen data in the PD dataset. The correlations and differences between ON and OFF states are not statistically significant, with the exception of the differences in ON/OFF state mean MDS-UPDRS-gait predictions when using the OpenPose library.

Conclusion

Based on our experiments, the addition of training data from the PD cohort worsened performance of the model significantly when evaluating on unseen examples from this dataset. It is hypothesized that the inclusion of data from a different clinical population which was collected in a different environment and annotated by different clinicians provided conflicting information to the model with respect to what a prototypical walk of each clinical score category should look like. We hypothesize that due to the differences in the labels/input data, data points from the smaller PD cohort served as outliers or anomalous points and provided conflicting information to the model which was otherwise trained on a larger cohort of datapoints from the DIP dataset. This suggests that proposed model is not able to identify and distill the identifying characteristics of each MDS-UPDRS-gait score class well when trained on a small number of examples from two different datasets. Interestingly, the range of scores predicted by the model was larger when the PD walks were included in the training set (Figure B-1 vs Figure A-1), suggesting that the model learned to make predictions at this higher range through the inclusion of examples of the walks with clinician-annotated scores of 3 available in the PD dataset. Future work will examine whether also training on walks from the dataset on which the model will be evaluated is beneficial when larger datasets are available.

Appendix C – Evaluation of OF-DDNet Model

In addition to the ST-GCN model presented in the main text, we explored the ordinal focal neural double-feature, double-motion network (OF-DDNet) proposed by Lu et al. [2]. This file presents the additional methods and results associated with this model.

Methods

As with the ST-GCN model, the OF-DDNet model was trained on the 2D joint trajectories extracted from the pose-estimation libraries (AlphaPose, Detectron, OpenPose). Similarly, the DIP dataset was used for training and the model was evaluated on the MDC dataset of adults with PD.

Additional File 1

Unlike for the ST-GCN model, the input data was not normalized as the internal features extracted by the OD-DDNet are location and viewpoint invariant [2]. However, the model was adapted to predict continuous UPDRS-gait scores by multiplying the predicted logits by the corresponding scores and summing for all scores.

Results

In our experiments, the "middle" weight configuration provided in the source code of the original DDNet work [3] yielded the best results and was thus used for the results presented below.

Figure C-1 presents the UPDRS-gait scores predicted by the OF-DDNet model when trained on the DIP dataset and evaluated on the PD dataset. The results are grouped by patient and clinical visit, and are presented during ON and OFF treatment conditions.

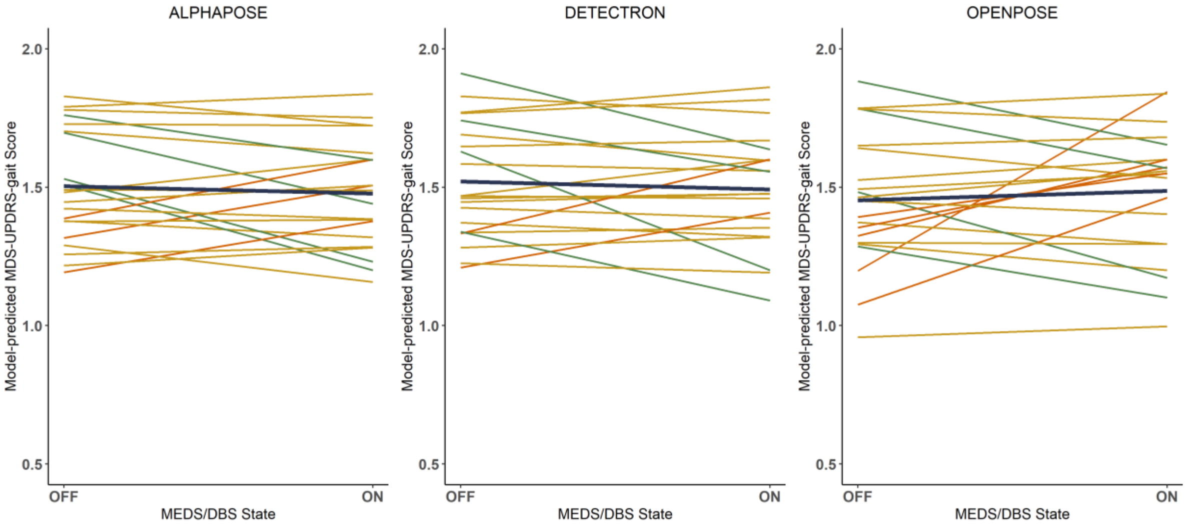

Figure C-1. Spaghetti plots of MDS-UPDRS-gait scores predicted by the model in ON and OFF states, grouped by patient and clinical visit. Green lines indicate paired walks where the MDS-UPDRS-gait score was higher in the OFF state than the ON state (indicating improvement in gait ON treatment), while red lines denote the pairs where the MDS-UPDRS-gait score was higher ON treatment (indicating worsening gait). Yellow is used to denote pairs where no change was noted between the two treatment conditions. The navy lines represent the mean prediction for each

Unlike the ST-GCN results presented in Figure 2 of the main text, there is no clear downward trend in UPDRS-gait scores predicted by the OF-DDNet when moving from OFF to ON treatment conditions.

Table C-1 presents the F1-scores for the OF-DDNet model when trained on the DIP dataset and evaluated on the PD dataset. The mean predicted scores during ON and OFF treatment conditions, as well as the significance values for a one-tailed paired t-test assessing whether the predicted OFF condition score is higher than the ON condition score for walks paired by participant and clinical visit is also presented in Table C-1.

TABLE C-1

Macro-averaged Precision, Recall, F1-Score, Mean MDS-UPDRS-Gait Score Prediction During ON and OFF States and Paired T-Test Significance Value for the OF-DDNet Model

Pose-estimation library	Precision of Rounded Predictions (mean ± STD)	Recall of Rounded Predictions (mean ± STD)	F1-score of Rounded Predictions (mean ± STD)	Mean predicted MDS-UPDRS-gait score – OFF treatment	Mean predicted MDS-UPDRS-gait score – ON treatment	Paired t-test p-value for difference in model predicted scores in ON/OFF states
**AlphaPose**	0.14 ± 0.04	0.23 ± 0.04	0.16 ± 0.04	1.50 ± 0.05	1.48 ± 0.13	.208
**Detectron**	0.19 ± 0.04	0.24 ± 0.03	0.18 ± 0.04	1.52 ± 0.07	1.49 ± 0.16	.211
**OpenPose**	0.13 ± 0.05	0.21 ± 0.03	0.15 ± 0.03	1.45 ± 0.07	1.49 ± 0.14	.756

Additional File 1

Consistent with the ST-GCN model, the F1-scores for the OF-DDNet model were low, indicating that the output of the model does not an accurately predict MDS-UPDRS-gait score. However, unlike for the ST-GCN model (Table 2, main text), the predicted OFF treatment scores were not significantly higher than the ON treatment scores for the OF-DDNet model.

Table C-2 presents Kendall's Tau-b correlation coefficient (τ_B) and p-value for significance of the correlation in the difference in UPDRS-gait scores as labelled by the clinician and by the OF-DDNet model.

TABLE C-2

One-tailed Kendall τ_B Estimates and P-Values for Correlation Strength Between Model-Predicted and Clinician-Annotated MDS-UPDRS-Gait Values and Differences in Scores Between ON and OFF States for the OF-DDNet Model

Pose-estimation library	Correlation of MDS-UPDRS-gait score predicted by ML model and annotated by clinicians		Correlation of difference between ON/OFF state as rated by clinicians and ML model	
	Kendall τ_B estimate	Kendall τ_B p-value	Kendall τ_B estimate	Kendall τ_B p-value
**AlphaPose**	0.02	0.443	0.09	0.298
**Detectron**	0.09	0.222	0.03	0.438
**OpenPose**	-0.08	0.750	0.17	0.152

Conclusion

In our experiments, the OF-DDNet model did not predict significantly lower UPDRS-gait scores during ON and OFF treatment states (Table C-1). The raw scores and differences between the scores predicted during ON and OFF treatment conditions as predicted by the OF-DDNet model were not correlated with the scores annotated by the clinicians only when any of the pose-estimation libraries were used (Table C-2). Unlike the ST-GCN model, the OF-DDNet model was not responsive to treatment condition and magnitude on the dataset evaluated in this study.

Appendix D – Evaluation ST-GCN by Repetition

When training the ST-GCN model evaluated in this work, large differences were noted in the raw MDS-UPDRS-gait values predicted by the model after training when initialized with different seeds (Figure D-1). For this reason, the model was trained from scratch five times (with different starting seeds) and the model performance in the main manuscript was reported using the mean prediction for each walk across all five repetitions. However, a measure of the reliability of the model is the stability of the main conclusion over each repetition. For example, robust and reliable model should yield the same conclusion (ie. identification of improvement, no change, or worsening parkinsonism in gait) when evaluated on each repetition.

Results and Discussion

Table D-1 presents the predicted direction of change for each repetition of the model, the mean model prediction (as reported in the main manuscript), the direction of change as annotated by each rater, as well as the mean clinician annotation in the ON and OFF state and direction of change for each paired walk.

From Table D-1, it was observed that the model-predicted direction of change was relatively stable across each model training repetition. For all paired walks, any deviation in the direction of change across different model training repetitions was between adjacent categories (ie. no row contained both a repetition indicating an "increase" and "decrease" in predicted MDS-UPDRS-gait score). Furthermore, in 13 of the 21 paired assessments, the model-predicted direction of change was consistent across all five repetitions. This suggests that even across different weight initializations and other stochasticity associated with training, the models are able to learn similar features of gait across repetitions such that the direction of their predictions on unseen walk pairs are relatively consistent. In our experiments, there was a difference in the number of pairs for which the model-predicted direction of change was congruent with the mean clinician direction of change (ranging from 11 to 13 out of the 21 pairs). The number of assessments where at least one of the repetitions of the model-predicted directions of change were congruent with the mean clinician-annotated direction is slightly higher at 16 out of 21.

From Table D-1, it can be observed that the five trials where the model predicted an increase in MDS-UPDRS-gait scores between the OFF and ON treatment condition corresponded to the trials which were not consistent with the mean

clinician-annotated direction of change. Of the five trials where the model predicted an increase in MDS-UPDRS-gait score, two of the trials had a mean clinician-annotated MDS-UPDRS-gait score of 3 (severe impairment). Individuals with this level of impairment were not present in the DIP training set, so it is likely that the model underestimated the level of parkinsonism in the OFF state, and thus always predicted a higher score in the ON state. This hypothesis is supported by Figure D-1, in which the red lines (representing an increase in MDS-UPDRS-gait score when moving from OFF to ON state) are generally on the lower end of the range in the OFF state. Furthermore, in one of the five trials where there was an increase in model-predicted score when moving from OFF to ON treatment state, one of the clinicians also noted an increase in their annotated MDS-UPDRS-gait score (Table D-1, fourth row from bottom).

Overall, the direction of change between ON/OFF states as predicted by the ST-GCN model is generally consistent between repetitions, suggesting the stochasticity of training does not impede the key features learned by the model.

Additional File 1

TABLE D-1

Direction of Change for Each Repetition of the ST-GCN Model Trained, Each Clinician, and Mean Clinician-Annotated MDS-UPDRS-gait Score

Person ID	Direction of Change in Model-Predicted MDS-UPDRS-gait Score Between OFF and ON States						Direction of Change in Clinician-Annotated MDS-UPDRS-gait Score Between OFF and ON States			Mean Clinician-Annotated MDS-UPDRS-gait Score by State	
	**Rep 1**	**Rep 2**	**Rep 3**	**Rep 4**	**Rep 5**	**Mean prediction across reps (presented in main text)**	**Rater 1 (Unblinded)**	**Rater 2 (Blinded)**	**Mean Rater Score**	**OFF State Mean Clinician Score**	**ON State Mean Clinician Score**
1	no change	no change	no change	decrease	no change	no change	no change	decrease	no change	1.5	1
2	decrease	decrease	decrease	decrease	decrease	decrease	decrease	decrease	decrease	1.5	0
3	decrease	no change	no change	no change	decrease	decrease	decrease	decrease	decrease	2.5	1.5
4	decrease	decrease	decrease	decrease	no change	decrease	no change	no change	no change	2	2
5	decrease	decrease	decrease	decrease	decrease	decrease	decrease	decrease	decrease	2	1
6	no change	decrease	decrease	decrease	decrease	decrease	no change	no change	no change	1	1
7	no change	decrease	decrease	decrease	decrease	decrease	decrease	decrease	decrease	3	1.5
8	decrease	decrease	decrease	decrease	decrease	decrease	decrease	decrease	decrease	2.5	0
9	no change	decrease	decrease	decrease	decrease	decrease	no change	decrease	no change	2	1.5
6	no change	no change	no change	no change	no change	no change	no change	decrease	no change	1	0.5
7	increase	increase	increase	increase	increase	increase	decrease	decrease	decrease	3	1.5
10	no change	no change	decrease	no change	decrease	no change	no change	decrease	no change	0.5	0
11	no change	no change	no change	no change	no change	no change	no change	no change	no change	0.5	0.5
8	decrease	decrease	decrease	decrease	decrease	decrease	decrease	decrease	decrease	1.5	0
12	decrease	decrease	decrease	decrease	decrease	decrease	decrease	decrease	decrease	3	1.5
1	increase	increase	increase	increase	increase	increase	no change	decrease	no change	1.5	1
4	no change	no change	increase	increase	increase	increase	decrease	decrease	decrease	3	1.5
9	increase	increase	increase	increase	increase	increase	increase	no change	no change	1	1.5
10	no change	decrease	decrease	decrease	decrease	decrease	decrease	decrease	decrease	1	0
11	no change	no change	no change	no change	no change	no change	decrease	no change	no change	0.5	0
13	increase	increase	increase	increase	increase	increase	decrease	decrease	decrease	1.5	0.5
	13	12	11	11	13	13	Count congruent with mean clinician prediction direction				
	Count congruent with mean model prediction direction across reps						13	11	13		

Additional File 1

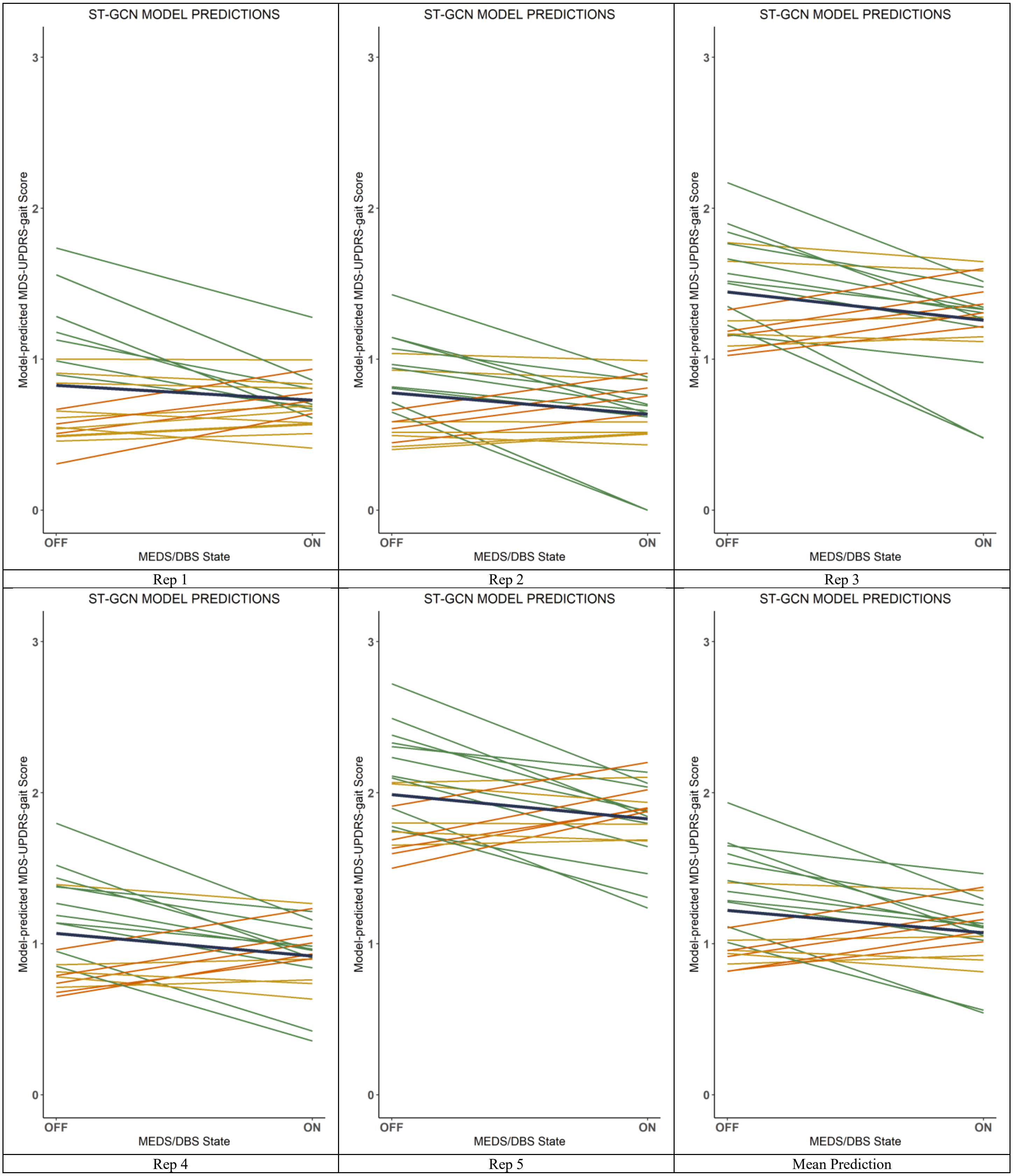

Figure D-1. Spaghetti plots of MDS-UPDRS-gait scores predicted by the model in the ON and OFF states, grouped by patient and clinical visit, and presented by model training repetition. Green lines indicate paired walks where the MDS-UPDRS-gait score was higher in the OFF state than the ON state (indicating improvement in gait ON treatment), while red lines denote the pairs where the MDS-UPDRS-gait score was higher ON treatment (indicating worsening gait). Yellow is used to denote pairs where no change was noted between the two treatment conditions. The navy lines represent the mean prediction for each treatment condition.

Additional File 1

Appendix E – Evaluation by Clinician Rater

In this study, two clinicians rated the videos on the MDS-UPDRS-gait scale independently. Rater 1 was not blinded to the ON/OFF state as they were present during data collection, while Rater 2 was not present during data collection and was blinded. In the main manuscript, we compare the results of the ST-GCN to the mean of the two clinicians' ratings. This section explores the differences in model performance when comparing to each rater separately and together.

Results

Table E-1 presents the one-tailed Kendall τ_B estimates of the correlation between the clinician and model predictions when comparing the raw values predicted, as well as the differences between the scores in the ON and OFF states. Of note, the raw model predictions are not correlated to Rater 1's annotations, while there are weak but statistically significant correlations between the model predictions and Rater 2 and the mean rater annotations. Interestingly, as seen in Figure E-1, the unblinded rater (Rater 1) assigned a higher score for one of the walks in the ON states than the corresponding walk in the OFF state. Rater 2, who was blinded assigned the same or lower score to individuals during the ON state than the OFF state for all assessments.

TABLE E-1

One-tailed Kendall τ_B Estimates and P-Values for Correlation Strength Between Model-Predicted and Clinician-Annotated MDS-UPDRS-Gait Values and Differences in Scores Between ON and OFF States by Clinician Rater

Rater	Correlation of MDS-UPDRS-gait score predicted by ML model and annotated by clinicians		Correlation of difference between ON/OFF state as rated by clinicians and ML model	
	Kendall τ_B estimate	Kendall τ_B p-value	Kendall τ_B estimate	Kendall τ_B p-value
**Rater 1 (unblinded)**	0.160	0.091	0.398	**0.014**
**Rater 2 (blinded)**	0.354	**0.001**	0.345	**0.024**
**Mean of Raters**	0.301	**0.004**	0.396	**0.010**

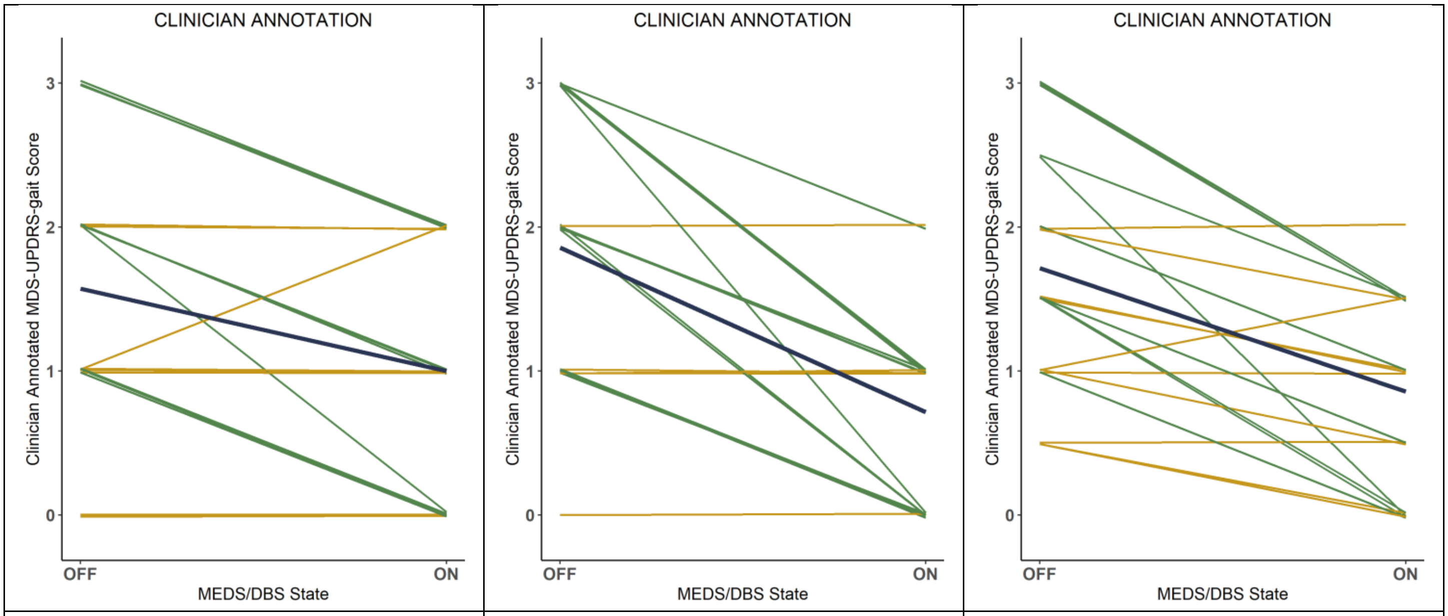

Rater 1 (unblinded) Annotations | Rater 2 (blinded) Annotations | Mean Clinician Annotations

Figure E-1. Spaghetti plots of MDS-UPDRS-gait scores labelled by the clinician in ON and OFF states, grouped by patient and clinical visit. Green lines indicate paired walks where the MDS-UPDRS-gait score was higher in the OFF state than the ON state (indicating improvement in gait ON treatment), while red lines denote the pairs where the MDS-UPDRS-gait score was higher ON treatment (indicating worsening gait). Yellow is used to denote pairs where no change was noted between the two treatment conditions. The navy lines represent the mean prediction for each treatment condition. Note that a small random jitter factor was applied to each clinician annotated score to improve the visualization.

The ICC between the two clinicians' ratings, as well as between each of the clinicians (and their mean assigned score) and the model predictions are presented in Table E-2.

Additional File 1

TABLE E-2
ICC Between MDS-UPDRS-gait Annotations from 2 Clinician Annotators, and between each Clinician Annotator and Model Predictions

	**ICC**	**p**	**95% CI**
ICC between annotations from 2 raters	0.727	**< 0.001**	[0.54, 0.84]
ICC between Rater 1 (unblinded) annotations and rounded model predictions	0.183	0.120	[-0.12, 0.46]
ICC between Rater 2 (blinded) annotations and rounded model predictions	0.306	**0.023**	[0.01, 0.56]
ICC between rounded mean clinician annotations and rounded model predictions	0.189	0.112	[-0.12, 0.46]

As seen in Table E-2, the ICC between the clinicians' rating and the model are significantly lower than between the two clinicians. The calculation of ICC requires the comparison of values belonging to discrete classes. For this reason, the model predictions were rounded to the nearest integer to match with the MDS-UPDRS-gait scale used by the clinicians. However, due to the small range of the model-predicted MDS-UPDRS-gait scores, most of the values rounded to 1. This limitation of the calculation of the ICC when paired with a model with a small prediction range results in the low ICC values noted in Table E-2.

References

[1] A. Sabo, S. Mehdizadeh, A. Iaboni, and B. Taati, "Estimating Parkinsonism Severity in Natural Gait Videos of Older Adults with Dementia," *IEEE J Biomed Health Inform*, p. 1, 2022, doi: 10.1109/JBHI.2022.3144917.

[2] M. Lu *et al.*, "Quantifying Parkinson's disease motor severity under uncertainty using MDS-UPDRS videos," *Med Image Anal*, vol. 73, p. 102179, 2021.

[3] F. Yang, Y. Wu, S. Sakti, and S. Nakamura, "Make skeleton-based action recognition model smaller, faster and better," in *Proceedings of the ACM multimedia asia*, 2019, pp. 1–6.
